# Median arcuate ligament syndrome (MALS): A case report of a young patient

**DOI:** 10.1016/j.ijscr.2025.111178

**Published:** 2025-03-19

**Authors:** Mindaugas Kiudelis, Matas Pažusis, Vytautas Kiudelis, Juozas Kupčinskas, Kristina Žvinienė

**Affiliations:** a Department of Surgery, Lithuanian University of Health Sciences, Kaunas, Lithuania; b Department of Gastroenterology, Lithuanian University of Health Sciences, Kaunas, Lithuania; c Department of Radiology, Lithuanian University of Health Sciences, Kaunas, Lithuania

**Keywords:** MALS, Celiac artery, Laparoscopic median arcuate ligament release, Median arcuate ligament syndrome, Minimally invasive surgery, Case-report

## Abstract

**Introduction:**

Median arcuate ligament syndrome (MALS) is a compressed celiac artery syndrome, also known as Dunbar syndrome. MALS is a clinically rare syndrome and is usually a diagnosis of exclusion. Diagnosis is a difficult task. Surgery is the treatment of choice, and laparoscopic surgery has promising results.

**Case presentation:**

A 40-year-old female with no comorbidities presented to her primary physician complaining of chronic abdominal pain, appetite loss, pain after eating, weight loss for about 5 years. Physical examination results were unremarkable. The abdomen/pelvis computed tomography angiography (CTA) was performed and 50% celiac artery stenosis was found. Due to inadequate symptom control and otherwise unremarkable workup, laparoscopic median arcuate ligament release was scheduled. The publication has been reported in line with the SCARE criteria [13].

**Clinical discussion:**

The etiology and pathophysiology of Dunbar syndrome are incompletely understood but may be related to both ischemic and neuropathic mechanisms. There are theories that MALS is associated with either a neurogenic or vascular origin for the clinical features, but objective evidence to support these theories is lacking. If MALS is suspected, abdominal/pelvic CTA can be used to verify the location of the celiac trunk. Despite the controversy regarding the pathophysiology, most patients with MALS have a good response to laparoscopic decompression.

**Conclusions:**

MALS is a rare and incompletely understood syndrome. Laparoscopic median arcuate ligament release and transection of the celiac plexus is an effective treatment for MALS.

## Highlights

• Rare Case of MALS: Presenting a unique case of MALS, which was diagnosed after a prolonged period of misdiagnosis.
• Diagnostic Approach: Use of advanced imaging techniques, including US, gastroscopy, colonoscopy, CT, which were key to identifying the condition.
• Surgical Treatment: Successful laparoscopic decompression of the median arcuate ligament, resulting in significant symptom relief.
• Long-term Outcome: Post-surgical follow-up reveals complete resolution of symptoms, supporting the efficacy of early surgical intervention in MALS.
• Clinical Relevance: Highlights the importance of considering MALS in patients with unexplained chronic abdominal pain and weight loss, leading to more accurate diagnosis and timely treatment.

## 1 Introduction

Median Arcuate Ligament Syndrome (MALS), also known as celiac artery compression syndrome or Dunbar syndrome, occurs when the celiac trunk is compressed by the median arcuate ligament. This compression leads to the narrowing of the celiac artery due to the thick fibrous tissue of the median arcuate ligament, which crosses anteriorly to the celiac trunk [1]. The fibrous ligament connects the diaphragmatic crura at the level of the aortic hiatus (T12-L1) and forms the anterior edge of the aortic foramina.

According to The European Society for Vascular Surgery guidelines, MALS is the most common cause of single vessel abdominal arterial stenosis [2].

A study by Katz-Summercorn et al. stated that in 92.6% the celiac trunk is adjacent to the median arcuate ligament (the distance could not be measured), and in 33.7% of those cases, the celiac trunk was compressed or distorted [3].

While the exact prevalence of MALS remains unclear, estimates suggest an incidence of about 2/100,000 patients per year, with a higher prevalence in females (4:1 ratio), and most commonly affects individuals between 30 and 50 years of age [3,4].

While there are no universally accepted diagnostic criteria for MALS, it is typically diagnosed through exclusion after ruling out other abdominal conditions like inflammatory bowel disease, peptic ulcers, and gallstone disease. Cusati et al. tried to evaluate the symptoms of MALS in their study consisting of 36 patients. This included abdominal pain (94%), postprandial abdominal pain (80%), weight loss (50%), bloating (39%), nausea and vomiting (55.6%), and exercise-induced abdominal pain (8%) [5]. MALS can lead to complications such as gastroparesis or pancreatic duodenal aneurysm due to chronic ischemia, collateral formation, and reduced blood flow [3,4]. Interestingly, stenosis of >50% of the celiac artery produced symptoms in only 1 of 8 patients in retrospective observational study of >1000 patients who underwent CTA (computed tomography angiography) [6]. An extensive workup to rule out more common gastrointestinal, hepatobiliary and pancreatic pathologies is recommended. This workup includes esophagogastroduodenoscopy, colonoscopy, motility studies, cross-sectional imaging, and hematologic studies, with CTA serving as an effective noninvasive diagnostic tool to assess arterial occlusion and rule out other intra-abdominal issues.

Although there is controversy surrounding the pathophysiology, the majority of patients with MALS show positive outcomes following laparoscopic decompression. This laparoscopic technique remains one of the most minimally invasive surgical options for treating MALS. There is another option for treatment - endovascular for patients who are unsuitable for traditional surgery or those who have not had a favorable response to laparoscopic decompression. This treatment usually involves using endovascular stents or angioplasty to address the vascular issues caused by the median arcuate ligament compressing the celiac artery. The objective of the endovascular procedure is to clear the blockage and enhance blood flow to the abdominal organs, thereby reducing the symptoms of MALS.

In our case report, we present a case of MALS successfully treated with laparoscopic median arcuate ligament release and celiac plexus transection in young female patient.

## 2 Case presentation

A 40-year-old female with no significant medical history presented with chronic abdominal pain, loss of appetite, postprandial pain, and weight loss over the past five years. She reported a reduction in food intake due to her symptoms and episodes of nausea and vomiting. She changed her eating habits and diet, but there was no result. She also stated that she had undergone extensive diagnostic workup in the past for the same symptoms but failed to reach a diagnosis. She was not on any medication, did not have allergies, and denied smoking or heavy alcohol consumption. Physical examination results were unremarkable, the patient's weight was 50 kg, height - 168 cm, and had a body mass index (BMI) of 17.72 kg/m^2^. Abdominal x-ray and abdominal ultrasound or duplex ultrasonography showed no significant findings.

## 3 Preoperative course

**Fig. 1:**
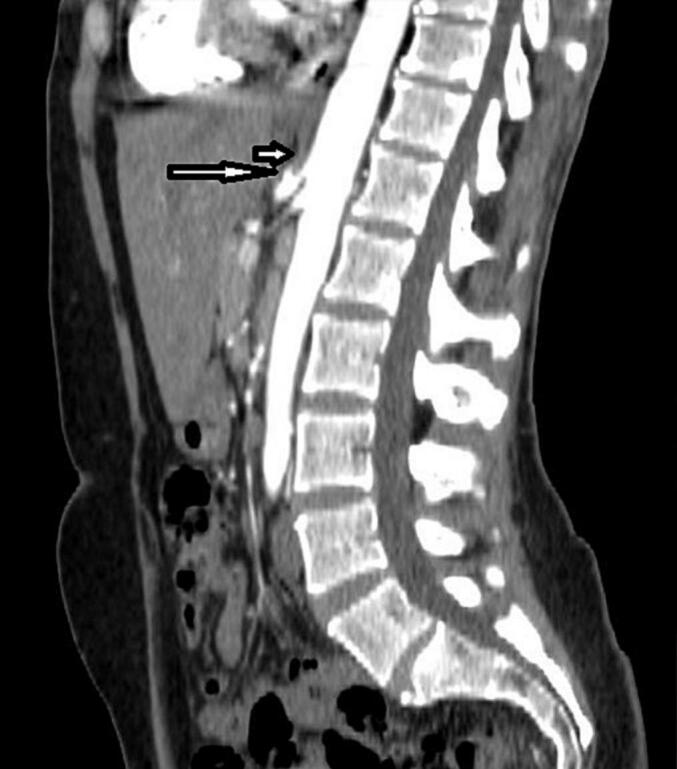
Sagittal reformatted, post intravenous contrast computed tomography. Long arrow demonstrates short segment stenosis of the coeliac trunk with a characteristic hooked appearance and post-stenotic dilatation. Short arrow demonstrates the thickening of the median arcuate ligament.

The patient referred to general surgeon in outpatient clinic for further evaluation and management. A gastroenterology consultation led to an upper endoscopy, revealing a small hiatal hernia and a negative *Helicobacter pylori* test. Colonoscopy showed no abnormalities. The abdomen/pelvis CTA was done and found 50% (mild) celiac artery stenosis (Fig. 1). Due to inadequate symptom control and otherwise unremarkable workup, laparoscopic median arcuate ligament release was scheduled in one month after the first visit to general surgeon.

**Fig. 2:**
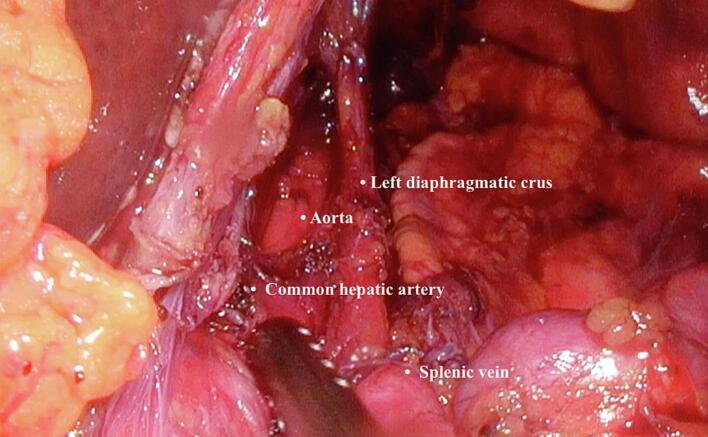
The celiac trunk is compressed by the medial arcuate ligament around its circumference. Incision of the ligament with a harmonic dissector to expose the root of the celiac trunk and the aorta. Final longitudinal view of the abdominal aorta.

During the operation, the surgeon stands between the legs, and the assistant stands on the right side of the patient. A camera port is inserted through the umbilical area, and four more ports are placed in the upper abdomen. A pneumoperitoneum blew 12 mmHg of gas into the abdomen. A Nathanson retractor was used to retract the left lobe of the liver. The left and right diaphragmatic crura are exposed to allow a good view of the surgical field before dividing the fibres of the medial arcuate ligament. The dissection is continued cranially to identify the branches of the celiac artery (common hepatic artery, left gastric artery, and splenic artery). The dissection is continued to the plane of the abdominal aorta. At this point, it is easier to recognize the connective tissue that consists of the median arcuate ligament, along with fibres from the celiac plexus. Using a laparoscopic harmonic dissector, the medial arcuate ligament fibres are cut. At the end of the dissection, the celiac trunk was free and not compressed. The patient tolerated the procedure well. (Fig. 2). Hospital stay was 3 days.

## 4 Postoperative course

**Fig. 3:**
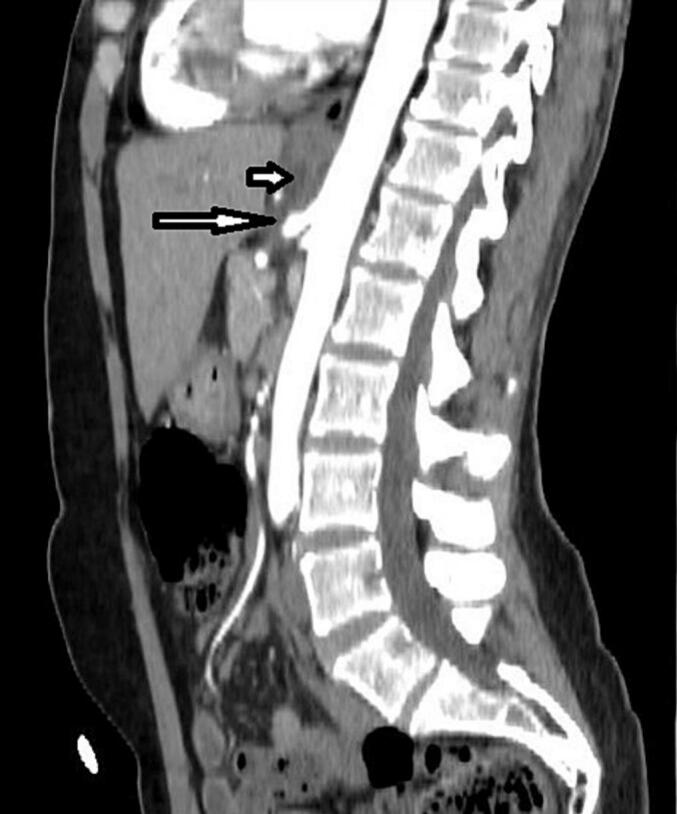
Sagittal reformatted, post intravenous contrast computed tomography. Long arrow demonstrates that the stenosis of the coeliac trunk was fully released. Short arrow demonstrates postoperative postoperative edema of tissues.

One month after the procedure, the patient visited the outpatient clinic of the Department of Surgery. The patient reported that she felt much better, gained weight, improved her appetite, and had no pain. The patient shows no postoperative complications or symptoms. Physical examination and history revealed no reported symptoms. Postoperative CT one month after surgery (Fig. 3) showed no celiac artery compression.

One year after the surgery, the patient was contacted by phone to inquire about her overall well-being post-operation. The pain had completely disappeared, and she no longer experiences the symptoms she had before the surgery. Her quality of life has improved compared to before the operation. A year after the surgery, a computed tomography (CT) scan was not repeated for the patient.

## 5 Discussion

MALS is a clinically rare syndrome and is usually a diagnosis of exclusion. Diagnosis is a difficult task. Although the incidence of MALS is not well known, it is more common in women aged 30–50 years (ratio 4:1) and in patients of thin build [1]. The etiology and pathophysiology of Dunbar syndrome are incompletely understood but may be related to both ischemic and neuropathic mechanisms. There are theories that MALS is associated with either a neurogenic or vascular origin for the clinical features, but objective evidence to support these theories is lacking.

It is usually characterized by chronic abdominal pain, nausea, vomiting, diarrhea and unintentional weight loss. The location of the pain is usually in the epigastrium. Physical examination may reveal epigastric tenderness or bruising that worsens with expiration [6]. In the case-report described by us, the patient had the same symptoms as described in the literature, such as nausea, pain after eating, and indigestion. However, no changes were found during the clinical examination of the patient.

An epigastric bruit is reported to be audible on auscultation in up to 35% of patients based on physical examination but is certainly not pathognomonic. These symptoms describe many diseases and syndromes throughout medicine, and MALS is certainly not the most common cause. Because of its rarity and relatively low prevalence, the radiologist may not always have this specific diagnosis in mind and therefore miss the characteristic findings on routine computed tomography (CT) [2]. If MALS is suspected, abdominal/pelvic computed tomography angiography (CTA) or magnetic resonance angiography (MRA) can be used to verify the location of the celiac trunk. Celiac artery decompression is reserved for symptomatic patients with evidence of celiac artery compression on inspiratory and expiratory vascular imaging studies. Asymptomatic patients should be advised to monitor postprandial abdominal symptoms [1]. The patient described in our case, as well as all the cases described in the literature, with abdominal pain of unknown origin, underwent all instrumental tests, such as endoscopy, colonoscopy, ultrasound, blood tests, but all tests were without changes. When CT scan angiogrphy was performed, pressure on the celiac trunk from the outside, and its stenosis by 50%, was diagnosed.

Despite the controversy regarding the pathophysiology, most patients with MALS have a good response to laparoscopic decompression. The laparoscopic surgical technique will continue to be considered one of the most minimally invasive surgical procedures for the treatment of MALS. Patients with MALS should preferably be examined and monitored in dedicated units [4]. As per existing data in the literature, we also performed a laparoscopic surgery to release the celiac trunk.

Surgery is the treatment of choice, and laparoscopic surgery has promising results. A common procedure is to separate the ligament fibres and other surrounding tissue around the origin of the celiac trunk to release compression. Compared with laparotomy, laparoscopic surgery can reduce surgical trauma and patient hospitalization, improve operative safety, and ultrasound can be used to confirm the opening of the celiac trunk [7,8]. Jimenez et al. analyzed the English literature on MALS surgery and laparoscopic surgery from 1963 to 2012 and reviewed postoperative outcomes, procedural details, and intraoperative and postoperative complications in 400 patients. The procedure was mainly medial arcuate ligament release; in some patients, celiac ganglions were removed or blood flow was restored in the celiac trunk. Results showed that 85% (339/400) of patients had postoperative symptom relief, with a recurrence rate of 6.8% (19/279) in laparotomy patients and 5.7% (7/121) when laparoscopic surgery was performed. Restoration of circulation did not improve symptoms compared with simple release of the medial arcuate ligament; the complication rate in the case of laparoscopy was 11.6%, in the case of open surgery - 6.5%. The most common complications of laparoscopy were bleeding and pneumothorax, postoperative complications were pancreatitis and gastroparesis (1 patient each), and bleeding required laparoscopy to be changed to open surgery in 9.1% of patients (11/121). The main complications of open surgery were postoperative vascular thrombosis, stroke, and gastroesophageal reflux. There were no procedure-related deaths from either procedure [3].

Endovascular treatment has shown potential in certain cases, particularly for patients with significant vascular involvement or those who have not responded to other treatment options. However, it is still regarded as a relatively new and less commonly used approach for MALS, and its long-term effectiveness and safety are still being studied [11,12].

The patient's postoperative period was smooth, abdominal pains after eating disappeared, increased appetite appeared, and the patient gained weight. Two months after the operation, the patient visited the outpatient department and had no complaints related to food or abdominal pain.

If symptoms of nausea, vomiting, postprandial pain, and weight loss persist even after laparoscopic ligament release, revascularization of the celiac artery by endovascular stenting or bypass can be considered as a secondary option [10]. The prognosis of MALS is generally good, given the high response rate to surgical decompression. The largest contemporary series reports an asymptomatic index of 75% with a mean follow-up of 9 years [9].

## 6 Conclusions

MALS is a rare but important cause of chronic abdominal pain and weight loss, and its diagnosis requires a high index of suspicion. Laparoscopic median arcuate ligament release and celiac plexus transection have proven to be effective treatments, as demonstrated in our case. This case underscores the need for differential diagnosis in patients with unexplained abdominal symptoms and highlights the successful outcomes of surgical management for MALS.

## Consent

The consent statement in the author form was signed.

## Ethical approval

The Kaunas Regional Biomedical Research Ethics Committee (protocol no. BE-2-13).

## Sources of funding

Own

## Declaration of competing interest

No conflicts of Interest.
